# Health-Related Quality of Life Among Community-Dwelling Older Hong Kong Adults: Protocol of a Longitudinal Cohort Study with Improved NGO Administrative Data

**DOI:** 10.3390/ijerph22111720

**Published:** 2025-11-13

**Authors:** Howard Haochu Li, Shicheng Xu, Vivian Weiqun Lou, Alice Ngai Teck Wan, Tammy Bik Tin Leung

**Affiliations:** 1 Sau Po Centre on Ageing, The University of Hong Kong, Hong Kong SAR, China; haochuli@hku.hk; 2 Department of Social Work & Social Administration, The University of Hong Kong, Hong Kong SAR, China; 3 Silver School of Social Work, New York University, New York, NY 10012, USA; sx2497@nyu.edu; 4 Aberdeen Kai-Fong Welfare Association, Hong Kong SAR, China; alicewan.nt@aka.org.hk (A.N.T.W.); tammyleung.bt@aka.org.hk (T.B.T.L.)

**Keywords:** health trajectories, health-related quality of life, community-dwelling older adults, social-ecological model, ageing-in-place, community health promotion, administrative data research

## Abstract

**Background**: Population ageing is a global challenge, prompting ageing-in-place policies in Hong Kong to support community-dwelling older adults while reducing healthcare costs. Yet, their impact on health-related quality of life (HRQoL) remains underexplored amid Hong Kong’s long life expectancy and growing older population. Traditional surveys are costly and time-consuming, while routinely collected registration data offers a large, efficient source for health insights. This study uses enhanced administrative data to track HRQoL trajectories and inform policy. **Methods**: This is a prospective, open-ended longitudinal study, enrolling adults aged 50 or older from a collaborating non-governmental organization in Hong Kong’s Southern District. Data collection, started in February 2021, occurs annually via phone and face-to-face interviews by trained social workers and volunteers using a standardized questionnaire to assess individual (e.g., socio-demographics), environmental (e.g., social support via Lubben Social Network Scale-6), biological (e.g., chronic illnesses), functional (e.g., cognition via Montreal Cognitive Assessment), and HRQoL (e.g., EQ-5D-5L) factors. A secure online system links health and service use data (e.g., service utilization like community care visits). Analysis employs descriptive statistics, group comparisons, correlations, growth modelling to identify health trajectories, and structural equation modelling to test a revised quality-of-life framework. Sample size (projected 470–580 after two follow-ups from a 2321 baseline) is based on power calculations: 300–500 for latent class growth analysis (LCGA) class detection and 200–400 for structural equation modelling (SEM) fit (e.g., RMSEA < 0.06) at 80% power/α = 0.05, simulated via Monte Carlo with a 50–55% attrition. **Discussion**: This is the first longitudinal HRQoL study in Hong Kong using enhanced non-governmental organization (NGO) administrative data, integrating social–ecological and HRQoL models to predict trajectories (e.g., stable vs. declining mobility) and project care demands (e.g., increase in in-home care for frailty). Unlike prior cross-sectional or inpatient studies, it offers a scalable model for NGOs, informing ageing-in-place policy effectiveness and equitable geriatric care.

## 1. Introduction

Population ageing is a global challenge, prompting the widespread adoption of ageing-in-place policies to support older adults in remaining at home [1]. These policies are assumed to preserve social connections while offering a cost-effective alternative to overburdened healthcare systems [2,3,4]. However, their impact on quality of life remains underexplored, with evidence suggesting that ageing-in-place does not inherently ensure independence or well-being, as health, mobility, and social challenges may compromise outcomes [5,6,7]. Ageing-in-place is the ability of older adults to live safely and independently in their own homes and communities, supported by services that maintain their health and quality of life rather than moving to institutional care [2]. Given the heterogeneity of ageing experiences, understanding individual health trajectories is critical to optimizing these policies’ benefits.

Quality of life, as defined by the World Health Organization, encompasses individuals’ perceptions of their position in life within the context of their culture and value systems [8]. To provide a more practical focus, Patrick and Erickson introduced the term “health-related quality of life” (HRQoL), which considers the impact of health, illness, and treatment on quality of life [9,10]. However, a clear research gap remains: current HRQoL studies among community-dwelling older adults are insufficient for evaluating ageing-in-place and guiding care because they typically concentrate on specific subpopulations and narrow outcomes [11,12,13], emphasize single risk factors rather than the interactions among multimorbidity, functional decline, and social context [14,15,16], and rely on resource-intensive, survey-based designs that are episodic and small-scale, limiting representativeness and the ability to track longitudinal trajectories and timely change [17,18,19,20,21].

Community care providers are crucial for monitoring health risks and delivering interventions to sustain HRQoL, but the variability and complexity of older adults’ needs demand comprehensive, longitudinal, multi-domain data that extend beyond single risk factors [14,15,16]. Traditional surveys rarely meet these requirements due to cost and operational burden [17,18,19,20,21]. In contrast, purposefully built member registration administrative data—collected longitudinally for service delivery with large volume—can directly overcome these limitations: they provide time-stamped, repeated observations across diverse older adults; capture service use and care processes (e.g., visit types, intensity, and referrals) that are actionable for providers; support the integration of clinical and social care records to enable multi-factor risk modelling; and are updated routinely, allowing near-real-time monitoring, trajectory analysis, and the evaluation of ageing-in-place interventions at scale [17,18,19,20,21].

In Hong Kong, where life expectancy reaches 82 for males and 88 for females [22], the 65+ population is projected to grow from 18.4% (1.32 million) in 2019 to 33.3% (2.52 million) by 2039 [22], intensifying HRQoL demands [23]. Common health needs include chronic illnesses (e.g., hypertension: 58.4%, diabetes: 14% [24]), mobility issues [24], depression (8.4% [24]), suicidal ideation (1.2%) [25], and social isolation (13% living alone [24,26]; 30.4% not participating in social activities [27]), driving the demand for community care. Since 1977, Hong Kong has prioritized ageing-in-place supported by NGO-led community care and, since 2019, District Health Centres (DHCs) to promote self-managed health. DHCs provide district-based services like chronic disease screening, health education, and rehabilitation to enable independent living and reduce institutionalization [28,29]. Although ageing-in-place is popular among older Chinese city-dwellers in Hong Kong, its long-term impact on HRQoL remains under-researched [30,31,32]. Community care providers, including District Health Centres and NGOs, routinely collect data on chronic conditions, health events (e.g., falls, hospitalizations), functional status, and service use during screenings [28,29]. These factors can serve as covariates to predict HRQoL trajectories.

This study will establish a structured longitudinal database with purposefully built member registration administrative data to address the insufficient knowledge regarding community healthcare needs in Hong Kong. Since the key limitation of administrative data is that it is not originally collected for research purposes [17], the research team co-designed a comprehensive assessment system with a collaborating NGO to support robust data collection and statistical analyses. The assessment system is also linked to the service utilization record of each registered member to examine service allocation efficiency. The study results will be shared with community members, who are viewed as having the best knowledge about improving the health of their community [33]. This community health promotion approach is expected to increase community members’ control over their health, address health inequalities, and enhance the well-being of the community [34].

## 2. Methods

### 2.1. Aims

This study has three specific objectives: (1) to establish a purposefully built member registration administrative database longitudinally tracking HRQoL trajectories among community-dwelling older adults; (2) to refine and validate a revised HRQoL model based on Ferrans et al. [10], integrating multi-level risk factors leveraged on the database; and (3) to assess the effectiveness of ageing-in-place policies on HRQoL by analyzing health trajectories and service needs. At the individual level, the database will identify risk factors and at-risk groups to enable early intervention. At the organizational level, linking service utilization records will optimize resource allocation and management. At the community level, the database will aggregate trajectories to anticipate social care (e.g., social support programmes) and long-term care demands (both in-home services like nursing/physiotherapy and institutional backup for severe decline), informed by HRQoL, medical needs (e.g., chronic conditions, health events via eHealth-linked records), functional status, and service utilization trends.

### 2.2. Conceptual Framework for Health-Related Quality of Life

**Figure 1:**
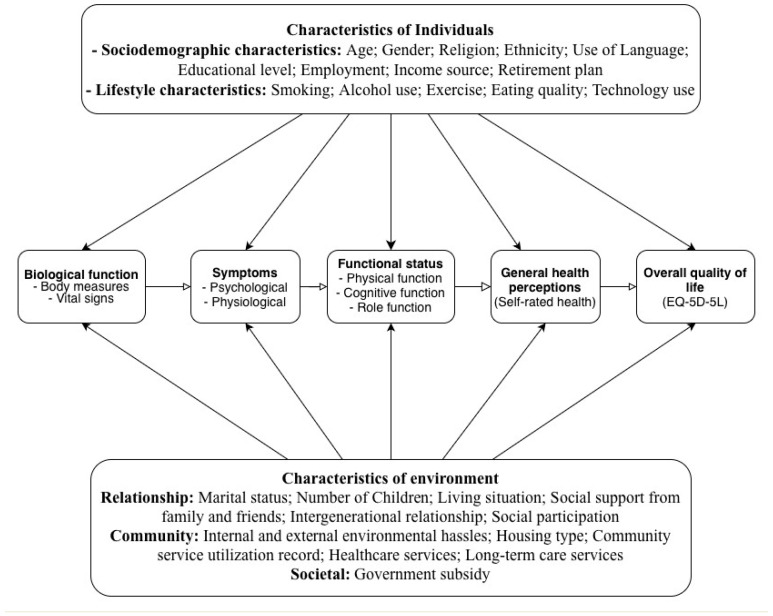
Conceptual model of health-related quality of life for community-dwelling older adults.

This study integrates McLeroy et al.’s social–ecological approach with Ferrans et al.’s revised HRQoL model to create a multi-level framework [10,35]. The social–ecological approach posits that ageing-in-place policies impact health variably due to individual and environmental interactions, making it ideal for examining longitudinal policy effects [35]. Ferrans et al. refine Wilson and Cleary’s model linking biological function, symptoms, functional status, general health perceptions, and HRQoL, with individual (e.g., demographic, psychological) and environmental (e.g., social, physical) influences explicitly defined [10]. McLeroy et al.’s model complements Ferrans et al.’s by expanding environmental characteristics into interpersonal and community levels, enabling rigorous analysis of multi-level influences on the HRQoL. Validated across conditions like HIV/AIDS [36], heart failure [37], and cancer [38], and predictive of mortality and service use in older adults [39], this model is robust [40,41]. We extend it by incorporating Zubritsky et al.’s ageing-specific additions (cognition, behaviour, and long-term services) and applying it to Hong Kong’s community-dwelling older population [42] (Figure 1). The revised HRQoL model is culturally adapted for Hong Kong’s older adults by incorporating variables like intergenerational relationships and caregiving stress, reflecting Confucian values, and using validated tools (e.g., EQ-5D-5L, CZBI-Short) tailored to Chinese older adults [43,44,45]. This ensures relevance in the context of family-centric care and urban ageing-in-place policies [30,31]. In Hong Kong’s Confucian-influenced culture and ageing-in-place policy (e.g., NGO care and DHCs), this integration captures how family/community supports moderate health pathways, addressing gaps in prior studies.

### 2.3. Study Design

This is a community-based, multicenter, prospective, open-ended longitudinal study with ongoing data collection via phone and face-to-face interviews in Hong Kong’s Southern District. At the time of registration for the membership (during the baseline visit), participants are encouraged to attend as many annual follow-up visits as possible.

### 2.4. Study Setting

The Southern District, one of Hong Kong’s 18 districts, has 263,278 residents (3.6% of the territory’s population), with 21.6% aged 65+—among the top five ageing districts [26,46]. Its post-intervention poverty rate is 6.1%, reflecting a socioeconomically diverse population with public (45.7% of Hong Kong residents in 2021) and private (53.7%) housing [47,48,49]. The Southern District’s high proportion of older population and housing diversity make it suitable, but its lower poverty rate (6.1% vs. Hong Kong’s 7.9%) and urban access to DHCs may have a potential to limit representativeness for some other districts [26,46,47]. Findings, however, remain transferable due to shared ageing-in-place policies and methodological adjustments [28,29].

### 2.5. Study Sample

This study will include as many eligible subjects as are willing to participate. Participants are members of a collaborating NGO, registering after February 2021, aged ≥50, and of any gender or ethnicity. Exclusion criteria include inability to provide informed consent (e.g., severe cognitive impairment). Members pay an annual fee of HKD 20–30 for services, waived for Comprehensive Social Security Assistance recipients. Follow-ups occur at annual membership renewals. The study sample differs demographically from the general Southern District population due to non-random, convenience sampling based on NGO membership, resulting in a sample skewed toward more socially engaged, older, female, and lower-income individuals. This will be addressed analytically. The sample size for this longitudinal cohort study was calculated to ensure sufficient statistical power to achieve its objectives: tracking HRQoL trajectories, validating a revised HRQoL model, and evaluating ageing-in-place policies among community-dwelling older adults in Hong Kong’s Southern District. Targeting a minimum baseline sample of 1600–2500 participants, the calculation accounts for key analyses, including latent class growth analysis (LCGA) and structural equation modelling (SEM), which require 300–500 and 200–400 participants, respectively, to detect small to moderate effect sizes (e.g., Cohen’s d = 0.3 for HRQoL changes) with 80% power and alpha = 0.05 [50,51]. The sample size (*n* = 2321 baseline, targeting 470–580 post-two follow-ups) assumes a small-to-moderate effect size (Cohen’s d = 0.3 for LCGA; standardized β = 0.2–0.3 for SEM) to detect HRQoL trajectory differences (e.g., EQ-5D-5L changes) and model relationships (e.g., social support to function) at 80% power, α = 0.05 [50,51]. Subgroup analyses by gender, age (50–64 vs. ≥65), chronic conditions, and housing type will target 100–150 participants per group to detect moderate effects, and weighted models will address sampling imbalances (e.g., female over-representation) [24,27]. Pilot-phase attrition at the first follow-up was 71% (670 of 2321 retained), driven by selective follow-up due to specific target requirements and manpower limits rather than true loss to follow-up. A reduced attrition rate of 50–55% (retention rate of 45–50%) was assumed for subsequent follow-ups, leveraging the NGO’s outreach services (e.g., regular contact and support programmes) to improve retention [52]. Although early attrition exceeded projections, improved retention in later cohorts supports a target of 470–580 retained participants, consistent with Hong Kong studies reporting a 20–30% annual loss in older cohorts [24]. To maintain 400 participants for LCGA/SEM after two follow-ups with 50–55% attrition per follow-up, the baseline sample was inflated (e.g., 400/(1 − 0.525)^2^ ≈ 1807, where 0.525 is the midpoint) [52]. The current enrollment of 2321 participants exceeds this requirement, yielding approximately 470–580 participants after two follow-ups, supporting robust subgroup analyses and accommodating heterogeneity in health needs. This sample size is justified by the need to capture diverse HRQoL trajectories, address gender imbalances through targeted male recruitment, and provide generalizable insights for policy in a rapidly ageing population, with ongoing open-ended recruitment further ensuring adequacy if attrition exceeds expectations [52].

### 2.6. Data Collection

Pilot data collection began in February 2021. By the end of 2024, 2321 participants completed baseline questionnaires, and 670 and 155 participants completed first and second follow-ups, respectively, with selective follow-ups due to specific target requirements and manpower constraints, not full attrition. Participants were prioritized based on engagement or data completeness, testing feasibility. Registered social workers conducted baseline interviews, with trained volunteers assisting in follow-ups. Follow-ups are hybrid (in-person preferred; phone if absent [53]), with loss after three contact attempts [27]. Renewal is voluntary and decoupled from surveys. To mitigate attrition, a concrete retention plan includes flexible data collection modes, personalized reminders, community-building activities, and family and proxy involvement [54,55]. Baseline written consent suffices annually, with no financial incentives but service priorities. Data are collected annually to track HRQoL trajectories. We will have at least three data points (baseline + 2 follow-ups) to estimate trajectories. Baseline interviews, conducted by trained social workers, take 30–45 min, while follow-ups (about 30 min) use skip-logic to reduce burden. Breaks and hybrid formats (in-person/phone) accommodate older participants.

### 2.7. Assessment and Measurement

Our collaborating NGO collects users’ records (e.g., demographics, subsidies), service utilization/health events (e.g., Home Support Service users: frequency of nursing visits, falls, and hospitalizations) in routine, which have been integrated with assessments to track HRQoL longitudinally. These administrative data can enhance HRQoL assessment, providing real-world context and enabling robust longitudinal analyses of health trajectories. A standardized questionnaire, informed by the conceptual framework, assessing variables across five domains (Table 1), was purposefully designed. The instruments used are culturally validated for Hong Kong Chinese participants, with data collection by Cantonese-speaking staff to ensure cultural sensitivity [25,56].

***Individual Characteristics***. Sociodemographic (e.g., age, gender, and education) and lifestyle factors (e.g., smoking, exercise) capture personal influences on HRQoL.

***Environmental Characteristics***. Environmental characteristics are assessed across three domains reflecting external influences on HRQoL, per the social–ecological framework: (1) relationship factors, including marital status, number of children, living situation, social support via LSNS-6 [25], intergenerational relationship [43], and social participation; (2) community factors, such as internal and external environmental hassles (e.g., home hazards, accessibility), housing type (e.g., public or private), and service utilization that is contingent on individual needs (e.g., recent hospital discharge requiring intensive nursing vs. routine medication management), quantified by frequency (visits/month), type (medical/social), and intensity (duration), linked to assessments like GDS-15 scores or fall history for tailored allocation; and (3) societal factors, specifically government subsidies (e.g., Comprehensive Social Security Assistance, Old Age Allowance, Old Age Living Allowance, Disability Allowance, and Mandatory Provident Fund). The LSNS-6, validated in older Chinese populations, measures the social network size and quality [25]. The attitudes toward young people questionnaire, adapted from Pinquart and Silvia [43], uses a 13-item semantic differential scale (7-point, scores 13–91, and higher = more positive), translated into Chinese via forward-and-backward methods, with good reliability in our prior study (Cronbach’s α = 0.787–0.823).

***Biological Function***. Height, weight with Body Mass Index (BMI), and blood pressure are measured at registration to monitor physical health.

***Symptoms***. The symptoms include the following: (1) psychological (depressive and anxiety symptoms, loneliness, and stressful events) and (2) physiological (self-reported chronic illnesses, sleep quality, falls, pain, and gait). Depressive symptoms are screened with the PHQ-2, followed by severity assessment using the GDS-15, both validated in Hong Kong [57].

***Functional Status***. Functional status includes the following: (1) physical function including ADL via Modified Barthel Index; IADL via Lawton’s scale, validated in Chinese [58]; walking ability; and assisted device use; (2) cognitive function using MoCA, validated in Hong Kong, with trained staff [56]; and (3) role function including volunteering via VFI and VSI [59] and caregiving via BAFFS, CZBI-Short, and PHQ-2 [44,57,60]. VFI assesses volunteer motives; VSI measures outcomes; and BAFFS, CZBI-Short, and PHQ-2 evaluate the caregiving burden and family function.

***General Health Perception***. General health perceptions (GHP) are assessed with a single self-rated item, “In general, how is your health?” scored on a 5-point Likert scale (1 = very good, 5 = very poor) with a lower score indicating more positive health perception [61].

***HRQoL*** (outcome variable). HRQoL is evaluated using the self-reported EQ-5D-5L, validated for Hong Kong Chinese [45]. It assesses five dimensions (mobility, self-care, usual activities, pain/discomfort, and anxiety/depression) on a 1–3 Likert scale (1 = no difficulty, 3 = extreme difficulty), converted to an index (negative = poor HRQoL, ~1 = no issues) [45]. It includes an EQ-VAS (0–100; 0 = worst, 100 = best health). Reliability is good with Cronbach’s α = 0.77.

### 2.8. Data Management and Storage

Participants are assigned unique IDs to anonymize and link questionnaire responses and service records. Data are entered into a custom online system with checks to prevent missing items and logic errors during interviews. They are stored on an encrypted, password-protected university platform (AES-256 standard) [62] and backed up daily on both the survey system and platform servers. Data management complies with Hong Kong’s Personal Data (Privacy) Ordinance (Cap. 486) [63].

### 2.9. Data Analysis

Data analysis begins with descriptive statistics to summarize demographic variables and sample characteristics. Normality of observed variables is assessed using mean, median, skewness, and kurtosis to ensure distributional assumptions for subsequent tests. Group differences are examined with independent-sample *t*-tests and analysis of variance (ANOVA), assuming normality and equal variances where applicable. Relationships among key variables (e.g., HRQoL, functional status, and symptoms) are explored using Pearson’s correlation coefficient. For longitudinal analysis, latent class growth analysis (LCGA) identifies subgroups with distinct HRQoL trajectories, capturing variations in initial levels and growth patterns over time, aligning with the study’s aim to track health trajectories. Multinomial logistic regression then determines factors influencing these trajectories, reporting relative risk ratios (RRRs) with 95% confidence intervals. Structural equation modelling (SEM) tests the revised HRQoL model, evaluating direct and indirect effects with fit indices (e.g., CFI, RMSEA) [64]. Contingencies are modelled as time-varying covariates in LCGA/SEM (e.g., post-discharge events predicting higher utilization in declining HRQoL trajectories). Missing data will be handled using the full information maximum likelihood (FIML) for LCGA/SEM, assuming missing at random (MAR), with Little’s test for missing completely at random (MCAR), and multiple imputation as a backup for non-MAR cases (e.g., health-related attrition) to ensure robust estimates [65]. We will assess normality (absolute skew ≤ 2, kurtosis ≤ 7) and, if violated, use MLR with bootstrapping (≥2000 resamples); LCGA selection will prioritize lower BIC/AIC, entropy ≥ 0.80, and significant VLMR/BLRT (*p* < 0.05) with parsimony and class sizes ≥ 5%; SEM fit will target CFI/TLI ≥ 0.95, RMSEA ≤ 0.06, and SRMR ≤ 0.08, with robust chi-square reported only; and WLSMV will be used for ordinals [50,65]. Analyses are conducted using RStudio, R version 4.3.2 (data processing), SPSS version 29 (descriptive and basic statistics), and Mplus 8 (LCGA and SEM).

## 3. Discussion

This study significantly advances the growing body of literature on the revised Wilson–Cleary HRQoL model by applying it to community-dwelling older adults in Hong Kong with heterogeneous healthcare needs, thereby broadening the understanding of the ageing process from a longitudinal perspective. To our knowledge, it represents the first longitudinal study among this population to utilize administrative data, offering an innovative and cost-effective solution for routinely collecting personal health data and systematically linking them with service utilization records. The longitudinal dataset generated through this approach enables researchers to predict diverse health trajectories and investigate the risk factors associated with each trajectory, addressing a critical gap in ageing-in-place research. Unlike large survey cohorts (SHARE/ELSA/CHARLS), this study uses NGO administrative data to reduce recall bias and create linkages to real-world events; though the sample is smaller, the approach is scalable across NGOs.

Building on the ageing-in-place framework outlined in the Introduction (e.g., NGO-led home-based care and District Health Centres’ preventive screenings [28,29]), the custom big data system developed for this study serves as a scalable model for other NGOs operating senior service units. In Hong Kong’s context, where policies emphasize subsidized in-home support (e.g., nursing, meal delivery) and community programmes to manage chronic conditions while reserving institutional care for severe cases, by modelling trajectories with multifaceted data (beyond HRQoL, including medical needs like chronic illnesses and prior events), the database projects community-level demands—e.g., increased in-home care for low HRQoL to sustain ageing-in-place—or institutional needs if trajectories indicate irreversible decline. This system supports further investigation into the impact, appropriateness, and effectiveness of ageing-in-place policies in Hong Kong, providing a comprehensive and data-driven framework. The study’s results will be used to advise Hong Kong’s Social Welfare Department and Health Bureau—forecasting care needs to guide District Health Centre expansion and subsidy allocation [28,29]—while co-developing interventions with NGOs through community forums and publishing to promote scalable ageing-in-place models [65]. Through these efforts, the study aims to enhance the quality of life for older adults and inform both policy development and practical improvements in geriatric care. Cultural adaptation of the HRQoL model, through validated tools and community engagement, ensures alignment with Hong Kong’s cultural (e.g., filial piety) and policy (e.g., DHC services) contexts, distinguishing it from prior studies [11,12,66].

Additionally, the study generates valuable insights into the operations of the eight data collection centres located in Hong Kong’s Southern District, facilitating effective service matching. Utilizing a social–ecological approach, individual needs are classified into three distinct levels—individual, interpersonal, and community—to guide service provision. This classification process allows for the identification of at-risk groups requiring early intervention and supports the tailoring of services to meet their specific needs, such as pain management, fall prevention, and chronic illness management. For organizational purposes, the study analyzes the strengths of each centre’s service offerings and explores areas for improvement using aggregated data, with a particular focus on critical health domains like pain management, fall prevention, and chronic illness management. By leveraging this comprehensive dataset, the study seeks to enhance the quality and effectiveness of services provided by these centres, ultimately improving the overall well-being of the community-dwelling older adults they serve.

The study also adopts a community participatory approach by sharing layperson-friendly data results with volunteers, equipping them with an enhanced understanding of community health risks. These volunteers play an active role in deciding appropriate intervention strategies and disseminating health messages to the broader community, fostering a collaborative environment. This approach serves as a vehicle for mutual learning between researchers and community members and promotes health equity by ensuring community input. The cyclical and iterative nature of this process, involving regular feedback loops such as annual community reviews, is designed to achieve a beneficial balance between research and actionable outcomes, fostering a long-term commitment to improving services for older adults. By actively involving community members, the study promotes community health and builds a more inclusive community framework. This participatory model not only empowers volunteers but also ensures that interventions are culturally relevant and tailored to the specific needs of the community, leading to more sustainable and impactful health outcomes [66].

### Methodological Considerations

Initial data reveals that over 70% of participants are female, reflecting their predominant use of community centres as a primary resource. To address the gender imbalance and improve representativeness, the collaborating NGO’s outreach team will intensify the recruitment of older men by tailoring messages to men’s health interests (e.g., cardiovascular health), training male volunteers as peer ambassadors, and engaging in venues frequented by older men (e.g., tea houses, parks, and chess clubs), while accommodating accessibility needs to encourage membership and participation. The results from this relatively affluent, urban district may not generalize to other places, so the sample is less representative than a territory-wide cohort; however, focusing on one district yields more detailed, context-specific insights into local ageing needs. This district-level analysis serves as an informative case study, offering valuable insights into local health dynamics. Moreover, the risks caused by the representativeness problem (e.g., skewed toward socially engaged, older, female, and lower-income people) can be addressed by several strategies, such as statistical adjustments, sensitivity analyses, targeted recruitment, and transparent reporting. Furthermore, the administrative data collection model employed in this study holds potential for adaptation and application to other communities across Hong Kong and beyond, contributing to a more complete understanding of population health when scaled up. Recognizing attrition as an inherent challenge in longitudinal social survey research, we will reduce loss to follow-up through the aforementioned structured retention plan. Moreover, the study’s emphasis on a district-level administrative data collection model helps manage this issue effectively. The collaborating NGO’s well-established and conventional outreach services, such as regular contact and support programmes, are expected to mitigate the impact of participant dropout by maintaining engagement over time. This approach leverages existing infrastructure to sustain participation rates. In conclusion, by addressing these methodological considerations—gender imbalance, limited generalizability, and attrition—the study enhances our understanding of the complex biological and psychological processes that influence HRQoL among community-dwelling older people from a longitudinal perspective. These adjustments ensure the provision of robust and actionable insights, informing future research, policy development, and practical applications in geriatric care.

## Figures and Tables

**Table 1:** Summery table of the variables of interest on the conceptual model.

Category	Variables	Instrument/Scale
**Characteristics of individuals**		
***Sociodemographic characteristics***	Age	Calculated from date of birth (years).
	Gender	Self-reported: Male; Female.
	Religion	Self-reported: None; Traditional Chinese belief; Christian; Catholic; Islam; Buddhism; Taoism; Other.
	Ethnicity	Self-reported: Chinese; Non-Chinese.
	Use of Language	Self-reported primary language: Cantonese; Mandarin; Hakka; Teochew; Hokkien; English; Other.
	Educational Level	Self-reported: None; Primary school; Middle school; High school; Post-secondary education.
	Employment	Self-reported current or pre-retirement status: Managers; Professionals; Paraprofessionals; Clerical support; Service and sales; Technical support; Machine operators; Non-technical workers; Skilled fish/agriculture workers; Between jobs; Unemployed; Self-employed; Family caregiver; Retired.
	Income source	Self-reported primary source: Comprehensive Social Security Assistance (CSSA); Old Age Allowance (OAA); Old Age Living Allowance (OALA); Disability Allowance (DA); Pension (pre-2000 public servants); Mandatory Provident Fund (MPF, post-2000); Family subsidy; Savings; Rental income; Annuity; Insurance; Reverse mortgage; Job salary; Unwilling to answer.
	Retirement plan	Self-reported: None; Government subsidy; Savings/Rent/Interest; Elderly home/Return to hometown; Annuity/Reverse mortgage/Insurance; Children’s support; MPF/Pension; Other.
***Lifestyle characteristics***	Smoking	Self-reported: “Do you smoke?” (1 = Daily; 2 = Occasional; 3 = Never).
	Alcohol use	Self-reported: “Do you drink alcohol?” (1 = Never; 2 = <2 times/month; 3 = 2–4 times/month; 4 = 2–3 times/week; 5 = >3 times/week).
	Exercise	Self-reported: “How many times do you exercise per week?” (Open numeric response).
	Eating quality	Self-reported: “Do you think you eat well?” (1 = Worse; 2 = Bad; 3 = Fair; 4 = Good; 5 = Great).
	Technology use	Self-reported: (1) “What technology do you use?” (Smartphone; Tablet; Smartwatch; Computer; None); (2) “If any, what for?” (Family contact; Online chat; News; Music; Games; Movies; Email; Other).
**Characteristics of environment**		
***Relationship***	Marital status	Self-reported: Unmarried; Married; Cohabiting; Widowed; Divorced/Separated.
	Number of children	Self-reported count of male and female children (Hong Kong and mainland China).
	Living situation	Self-reported cohabitants: Alone; Parents; Spouse; Children; In-laws; Grandchildren; Other relatives; Domestic helpers; Friends; Other.
	Social support	Lubben Social Network Scale–6 (LSNS-6), validated for older Chinese.
	Intergenerational relationship	Self-reported: (1) “Did you join intergenerational activities?” (Yes/No); (2) attitudes toward youth (13-item semantic differential scale, 7-point, 13–91 range).
	Social participation	Self-reported activities: None; Educational; Hobbies/Leisure; Association volunteering; Other volunteering; Current events; Religious; Intergenerational; Social gatherings/Competitions; Health services; Outdoor; Exercise; IT/Tech use; Other.
***Community***	Internal environmental hassles	Self-reported home hazards: None; Slippery ground; Uneven surfaces; Poor lighting; Debris; Unstable furniture; Other.
	External environmental hassles	Self-reported external barriers: None; No elevator; Stairs; Slopes; Embankments; Distant locations; Other.
	Housing type	Self-reported: (1) Type (Public; Home Ownership Scheme (HOS); Private; Subdivided flat; Non-residential; Temporary; Dormitory; Elderly home; Other; Unwilling to answer); (2) address details (Street, Building, Floor, Unit).
	Long-term care services	Self-reported: (1) Use or waitlist status for Community Care Services (CCS)/Residential Care Services (RCS); (2) healthcare service use (Yes/No).
***Societal***	Government subsidy	Self-reported receipt: CSSA; OAA; OALA; DA; MPF (Yes/No for each).
**Biological function**		
***Body measures***	Height and weight	Measured: Height (cm); Weight (lb); Body Mass Index (BMI) calculated as (kg/m^2^).
***Vital signs***	Blood pressure	Measured: Systolic (mmHg); Diastolic (mmHg).
**Symptom**		
***Psychological symptoms***	Depressive/anxiety symptoms	Patient Health Questionnaire-2 (PHQ-2) for screening; 15-item Geriatric Depression Scale (GDS-15) for severity.
	Loneliness	Self-reported: “In the past 2 weeks, did you feel lonely?” (1 = Never; 2 = Certain situations; 3 = Sometimes; 4 = Often; 5 = Daily).
	Stressful event	Self-reported: “Did you have a significant stressful event?” (Yes/No).
***Physiological symptoms***	Chronic illnesses	Self-reported: None; Chronic obstructive pulmonary disease (COPD); High cholesterol; Heart disease; Hypertension; Asthma; Stomach disease; Diabetes; Cognitive impairment; Eye disease; Stroke; Parkinson’s; Depression; Arthritis; Osteoporosis; Gout; Ear, nose, and throat (ENT) disease; Cancer; Other.
	Sleep quality	Self-reported: “How’s your sleep quality?” (1 = Very good; 2 = Good; 3 = Fair; 4 = Poor; 5 = Very poor).
	Falls	Self-reported: (1) “In the past 12 months, how many times did you fall?” (Numeric); (2) “Where?” (Living room; Corridor; Toilet; Bedside; Kitchen; Outside; Other).
	Pain	Self-reported: (1) “In the past 3 months, did you have pain?” (Yes/No); (2) “If yes, severity?” (Pain Rating Scale, 0–10).
	Gait	Timed Up and Go Test (seconds).
**Functional status**		
***Physical function***	Activities of daily living (ADL)	Modified Barthel Index (1 = Independent; 2 = Needs assistance; 3 = Dependent).
	Instrumental ADL (IADL)	Lawton’s IADL Scale, validated in Chinese (1 = Independent; 2 = Needs assistance; 3 = Dependent).
	Walking abilities/devices	Self-reported: No difficulty/no aids; Difficulty/no aids; Cane/umbrella; Mobility aids (tripod/quad cane/frames); Manual wheelchair; Electric wheelchair; Other.
***Cognitive function***	Cognitive status	Montreal Cognitive Assessment 5 min (Hong Kong version) categorized as follows: Normal; Mild neurocognitive disorder; Mild cognitive impairment; Major neurocognitive disorder.
***Role function***	Volunteer experience	Self-reported: (1) Training (Yes/No); (2) Experience (Yes/No); (3) Skills (e.g., language, sports); (4) Volunteer Functions Inventory (VFI); (5) Volunteer Satisfaction Inventory (VSI).
	Caregiving status/stress	Self-reported: (1) Grandchildren care (Yes/No); (2) Older family care (Yes/No); (3) Domestic helpers (Yes/No); (4) Care hours/week (Numeric); (5) The Brief Assessment of Family Functioning Scale (BAFFS); (6) ZBI-4; (7) PHQ-2.
**General health perception**	Self-rated health	Self-reported: “In general, how is your health?” (1 = Very good; 2 = Good; 3 = Fair; 4 = Poor; 5 = Very poor).
**Health-related Quality of Life**	HRQoL	European Quality of Life–5 Dimensions (EQ-5D-5L) (1 = No difficulty; 3 = Extreme difficulty).

## Data Availability

No new data were created or analyzed in this study. Data sharing is not applicable to this article.
